# Assessing Perceptions Toward Aquatic Animal Welfare: A Study on the Perspectives of Educators, Students and Aquaculture Industry Stakeholders in South and South-Central Vietnam

**DOI:** 10.3390/ani16010026

**Published:** 2025-12-22

**Authors:** Sasha Saugh, Pham Kim Long, Lien-Huong Trinh, Oanh Duong Hoang, Huong Huynh Kim, Pham Van Day, Men Nguyen Thi, Simão Zacarias, Chau Thi Da

**Affiliations:** 1 Aquaglobal Veterinary Consulting Pty Ltd., Durban 4037, South Africa; support@aquaglobalvet.com; 2 College of Engineering and Technology, Tra Vinh University, 126 Nguyen Thien Thanh Street, Hoa Thuan Ward, Vinh Long 87000, Vinh Long Province, Vietnam; kimlong_phm@tvu.edu.vn; 3 Shrimp Welfare Project Ltd., International House, 45-55 Commercial Street, London E1 6BD, UK; lienhuong@shrimpwelfareproject.org; 4 School of Agriculture and Aquaculture, Tra Vinh University, 126 Nguyen Thien Thanh Street, Hoa Thuan Ward, Vinh Long 87000, Vinh Long Province, Vietnam; dhoanh@tvu.edu.vn (O.D.H.); hkhuong77@tvu.edu.vn (H.H.K.); phvday@tvu.edu.vn (P.V.D.); 5 School of Economics and Laws, Tra Vinh University, 126 Nguyen Thien Thanh Street, Hoa Thuan Ward, Vinh Long 87000, Vinh Long Province, Vietnam; nguyenmen20@tvu.edu.vn; 6 Institute of Aquaculture, University of Stirling, Stirling FK9 4LA, UK; 7 Group of Applied Research in Advanced Materials for Sustainable Development, Faculty of Applied Sciences, Ton Duc Thang University, Ho Chi Minh City 70000, Vietnam

**Keywords:** aquatic animal welfare, perception gap, education reform, aquaculture stakeholders, Vietnamese aquaculture

## Abstract

This study investigated the perceptions of AAW among educators (n = 47), students (n = 359), and aquaculture sector stakeholders (n = 34) in key aquaculture regions of South and South-Central Vietnam. Using a mixed-methods survey, perception scores, understanding of core welfare concepts, curricular coverage, and willingness to pay (WTP) for higher-welfare products were assessed. Although perception scores were high across surveyed groups, only 5.57% of students identified most of the Five Freedoms of Animal Welfare, while 17.0% showed no understanding of welfare. This highlighted a gap between awareness and understanding, leading to the development of the Awareness Depth Gap Framework. A small but statistically significant negative correlation (Kendall–Theil –Sen slope = −0.28, *p* < 1.25 × 10^−12^) indicated that greater AAW curricular inclusion was associated with stronger welfare attitudes. Willingness to pay modest premiums (6–10%) for higher-welfare products increased non-linearly with higher perception scores among educators and students. In contrast, aquaculture sector stakeholders appeared to prioritise economic factors over welfare. These results highlight the need for structured AAW curricula and community collaborative education to close knowledge gaps. This study provides the first empirical insights into AAW perceptions across the education and aquaculture sectors in Vietnam.

## Simple Summary

Vietnam’s aquaculture sector faces a major skills and knowledge gap. Many tertiary institutions still use outdated curricula and have limited resources, leaving graduates poorly equipped to apply aquatic animal welfare (AAW) in practice. Because of weak enforcement, low penalties, and a lack of species-specific guidelines, welfare standards are seldom implemented. Simultaneously, poor handling, overcrowding, and transport conditions persist on farms. Financial and cultural priorities often outweigh welfare concerns, so improving AAW requires education that links animal welfare to income, food security, and livelihoods. This study surveyed educators, students, and aquaculture professionals in South and South-Central Vietnam to assess their knowledge and attitudes toward AAW. Although most respondents showed concern for animal welfare, only about 6% of students could identify key welfare principles, and 17% showed no understanding at all. Educators and students were willing to pay 6–10% more for higher-welfare products, whereas aquaculture sector stakeholders prioritised profit. These findings reveal broad theoretical support but limited practical understanding. Structured AAW training, including community benefits, is essential to strengthen education, policy, and practice toward global welfare standards.

## 1. Introduction

Vietnam, a lower-middle-income country, faces challenges in tertiary education such as limited capacity, uneven quality, and a misalignment between graduate skills and market demands [1]. This study focuses on South and South-Central Vietnam, regions central to the country’s aquaculture industry, which account for ~90% of Vietnam’s aquaculture production area [2]. Despite being the world’s fifth-largest producer of farmed fish and the third-largest seafood exporter [3], Vietnam performs poorly in animal welfare legislation, earning an “F” grade on the World Animal Protection’s global index [4]. Existing legislation is largely awareness-driven, with weak penalties, limited enforcement mechanisms, and few species-specific guidelines aligned with WOAH standards [5]. The mismatch between production scale and welfare governance highlights the need to understand how aquaculture stakeholders perceive AAW and how prepared they are to support welfare-focused reforms.

Animal welfare describes the state of an animal in relation to its nutritional, environmental, health, behavioural, and mental needs [6,7], and for aquatic species, this involves their unique biological and husbandry requirements. Public perceptions also influence willingness to adopt welfare practices, and misconceptions or knowledge gaps can hinder progress [8]. While factors such as gender, age, religion, and personal experience often affect attitudes towards animal welfare [9,10], these patterns are primarily documented in Western contexts. Little is known about how such factors shape AAW perceptions in Vietnam, where cultural, economic, and industry-specific conditions may produce different trends. This gap is important given that Vietnamese employer reports that university graduates lack key competencies required in the job market [10]. Identifying whether an AAW education gap exists, and whether it affects students’ and educators’ perceptions, can guide curriculum development and support ethical, sustainable aquaculture. Perception surveys capture what people think, feel and do, providing insight into AAW interventions that are practical, widely accepted, and effective. This understanding informs not only education and training but policy reforms, technical support, and infrastructure improvements. It also supports the effective adoption of welfare practices, helping to manage market risks while meeting consumer expectations, retailer requirements, and certification standards.

Despite the central role of aquatic animals in Vietnam’s aquaculture economy, no studies have examined AAW perceptions or educational preparedness among university students, educators, or stakeholders. This exploratory study is the first to address this gap and evaluate whether an AAW education gap at the undergraduate level negatively impacts students’ perceptions towards AAW (hypothesis). The findings aim to inform national curriculum improvement in Vietnam, support policy development, and provide a model for other countries seeking to align aquaculture practices with international welfare standards. Although the authors recognise the importance of animal welfare to all animals, aquatic animal welfare is the focus of this study.

## 2. Materials and Methods

### 2.1. Study Area and Data Collection

The project covered South and South-Central Vietnam, including key academic and vocational institutions across multiple provinces in the Mekong Delta and coastal regions.

These included Bạc Liêu College of Economics and Technology and Bạc Liêu University (Bạc Liêu Province); Cà Mau Community College (Cà Mau Province); Đồng Tháp Community College (Đồng Tháp Province); Sóc Trăng Vocational College (Sóc Trăng Province); Southern Agricultural College (Hậu Giang Province); TràVinh University (TràVinh Province); and Cần Thơ University (Cần Thơ City) in the Mekong Delta. It also included Ho Chi Minh City University of Agriculture and Forestry (Ho Chi Minh City, Southeast) and Nha Trang University (Khánh Hòa Province, South Central Coast). Together, these institutions provide comprehensive coverage of the region’s aquaculture educational landscape.

**Figure 1:**
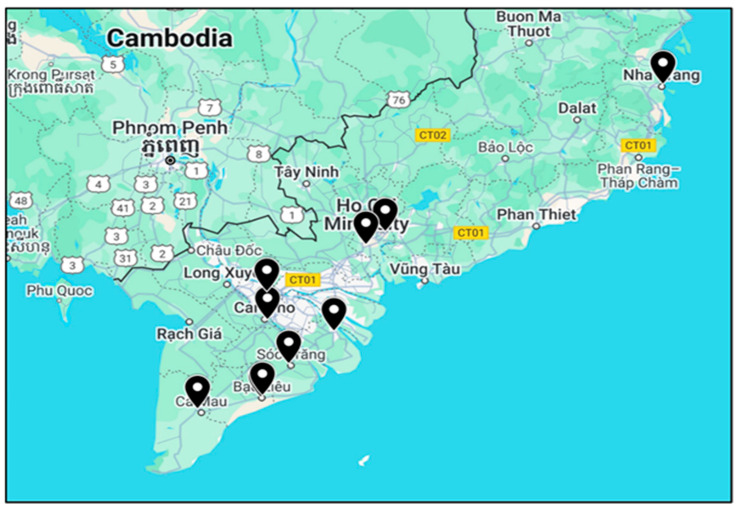
Map of tertiary institutions sampled in South and South-Central Vietnam for the aquatic animal welfare perception survey highlighted by black symbols.

Survey design and implementation: a mixed-methods survey was undertaken on a target population of 2048 students and 164 educators across ten universities and eight colleges in Vietnam, focusing on institutions offering aquatic animal or aquaculture-related programmes (Figure 1). Student surveys were conducted digitally (via Google Forms), while educators and aquaculture sector stakeholders (hereafter referred to as “stakeholders”) were interviewed telephonically. Google Forms Interview data were recorded digitally, and consistent interview procedures were followed. Survey instruments were developed, validated, and piloted prior to deployments, and reliability indices were provided for all survey instruments (Supplementary Materials Section S1).

Sampling strategy: A weighted, stratified random sampling approach [11] was employed for students, with educator samples proportionate to institutional student numbers (Supplementary Table S1) from the target population. Stakeholders were selected through convenience sampling within the TràVinh University (TVU) network. The study included an equal number of institutions (five universities and five colleges) to ensure balanced coverage of the educational landscape. Students (n = 359) were sampled in multiple stages: first by institution, then by major, and finally by academic year, with a 95% confidence level and a 5% margin of error. This ensured that each subgroup of students, across year level, major, and academic institution, was represented in proportion to its size, producing a more accurate, unbiased, and representative sample of the target population. Educators (n = 47) were randomly sampled, and the stakeholders (n = 34) sampled represented commercial enterprises, government agencies, and research institutions. The details on the inclusion criteria are provided in Supplementary Materials Section S2.

Survey instruments and variables: survey instruments assessed demographic characteristics, perceptions of AAW, curriculum exposure, and perceived education gaps. Variables investigated across respondent groups included common elements such as demographic factors and group-specific variables (Supplementary Materials Section S3). Survey instruments included 29 questions for students, 26 for educators, and 25 for stakeholders. A combination of Likert-scale, multiple-choice, binary, matrix, open- and closed-ended question formats was used. Welfare scoring: perceptions on AAW were evaluated using ten tailored questions, whilst their understanding of what AAW were scored based on the recognition of humane treatment, ethical and legal responsibility, the Five Freedoms [12]; and relevance of welfare across diverse interactions with aquatic animals.

Perception scores were categorised from low to very high and reflected knowledge, attitudes, and ethical awareness towards AAW. The details of the questions are described in Supplementary Materials Section S4 and Supplementary Tables S2–S4. Education gap scoring: four aspects of student perceptions of educational adequacy were assessed, including confidence in discussing AAW, curriculum sufficiency, satisfaction with materials, and teaching quality. A 40-point scale categorised education gaps from very low to high (Table 1), with higher scores indicating greater perceived deficits.

### 2.2. Data Analysis

Statistical analyses were performed in R-Studio (version 4.1.3). Normality and homogeneity were tested using the Shapiro–Wilk and Levene’s tests, respectively. Based on distributional results, Welch’s ANOVA was used for group comparisons on approximately normally distributed data with unequal variances. When normality assumptions were violated, the Kruskal–Wallis test was used. Both these tests provided robust analysis in the presence of unequal sample sizes, as an alternative to the classic ANOVA. The Kendall–Theil–Sen test was used for trend analysis due to its robustness to outliers and no requirement of normality. Bonferroni post hoc corrections were used where relevant. Results were reported as mean ± 1 SD. Perception and education gap scores were standardised to a 100-point scale. Significance was defined at *p* < 0.05. Weighted sampling and robust statistical methods ensured that results were representative and valid despite unequal sample sizes and variances.

Dependent variables included perception and education gap scores. Key independent variables, academic major and year, were analysed using Welch’s ANOVA to address unequal sample sizes. Other explanatory variables evaluated included gender, age, household income, religion, professional rank, highest educator qualification, teaching experience, seafood consumption, and welfare product mark-ups. ANCOVA was used to explore interaction effects.

## 3. Results

### 3.1. General Information on the Demographics of Survey Participants

Table 2 summarises the demographic characteristics of the survey participants, including gender, age, religion, monthly income, and teaching experience. Among participants, the majority were male (55.2%), while 43.9% were female, and <1% of participants preferred not to disclose their gender. Among students (n = 359), the majority were between 18–21 years old (61.6%), followed by 22–25 years old (37.3%), while very few were in the older age groups. Most students identified as non-religious (66.0%), with 24.0% identifying as Buddhist, and smaller proportions identifying as Catholic (4.26%) or following other religions (5.85%). Regarding income, most students reported less than 10 million VND (69.9%), with decreasing proportions in the higher income brackets.

The largest proportion of educators (66.0%) was aged between 36–45 years old, followed by those aged 46–55 years (29.8%). Most educators also identified as non-religious (76.6%), and the majority had over 15 years of teaching experience (63.8%). The monthly income varied, with the largest group (61.7%) earning between 10–20 million VND, and smaller proportions in the higher income brackets (Table 2). For stakeholders (n = 34), 55.9% were aged 36–45, followed by 23.5% aged 25–35, and 11.8% aged 46–55. Like the other groups, the majority (70.6%) reported no religious affiliation. Income distribution was broader compared to educators and students. About 32.4% earned 10–20 million VND, 23.5% earned 20–30 million, and 20.6% earned above 30 million (Table 2). The notably lower income among students compared with educators and stakeholders, aligns with welfare-related behaviours, including reduced engagement or limited ability to priorities welfare practices.

**Figure 2:**
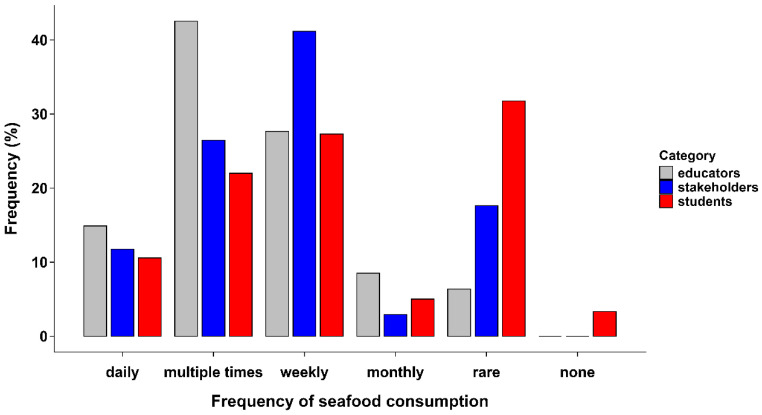
Illustration showing seafood consumption frequency among perception survey respondents (students (n = 359), educators (n = 47), and stakeholders (n = 34)) in South and South-Central Vietnam.

Figure 2 illustrates the frequency of seafood consumption among students, educators, and stakeholders. Most educators consumed seafood multiple times per week (over 40%), followed by weekly (approximately 30%), showing high consumption rates. Among stakeholders, consumption was primarily weekly (42%) or multiple times per week (25%). In contrast, students reported much lower seafood intake, with the majority consuming seafood on a weekly (29%) or rare (33%) basis. Furthermore, the frequency of aquatic animal consumption showed no significant relationship with perception scores. This pattern corresponds with varying levels of exposure to aquaculture environments. Students who have less direct industry experience and lower average income, appear to approach animal welfare in a more theoretical manner. In contrast, educators and stakeholders, who work closely with production and regulatory systems, tend to view welfare in relation to market demands and production efficiency. These differences reflect how seafood is normalised within each group.

### 3.2. Students’ Perspectives on AAW

Among students, 98.1% had no prior tertiary education, and their career aspirations were diverse but did not reference AAW. While most students (89.7%) recognised AAW education as beneficial for their careers, they did not prioritise it in their career plans. This reflects their awareness that immediate job opportunities in aquaculture rely more on foundational production skills rather than on specialised knowledge in AAW. This aligns with industry demands that emphasise practical management and operational competency over welfare expertise. Students cited benefits such as enhanced employment prospects and contributions to conservation, sustainability, welfare policy, and research. Most students (68.0%) regarded the current inclusion of AAW in the curriculum as adequate, while only 6.13% reported dissatisfaction. The remaining students were unsure (5.85%), were indifferent (1.39%), or did not answer because they had not studied it yet (18.7%).

**Figure 3:**
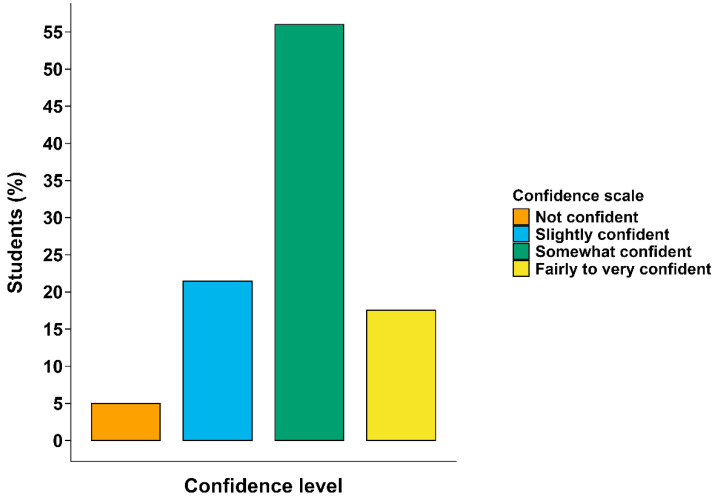
Confidence ratings of Vietnamese tertiary education students (n = 359) in discussing various components of aquatic animal welfare including, nutrition and feeding, pain, health and disease, natural behaviour, habitat needs, emotional states, environmental enrichment, and ethics and social values revealed that the majority (55.9%, n = 201) of students were somewhat confident.

Only 34.6% of students expressed a desire for improved AAW content (some made multiple recommendations), whilst 20.3% showed no preference for further inclusion. This highlights the importance of raising student awareness about career relevance of AAW. Satisfaction with learning resources and the overall learning experience was generally high, with 37.1% of students “very satisfied” and 40.7% “relatively satisfied”. Similarly, 48.2% were “very satisfied” with AAW teaching quality, communication skills, student support, and knowledge on AAW at their institutions, while 31.8% were “relatively satisfied”. However, only 17.6% expressed high confidence (“fairly to very confident”) in discussing AAW topics, with an average confidence score of 63.7% (Figure 3). With a minority of students (12.8%) requesting additional practical work, and over half (56.0%) reporting that they felt “somewhat confident” the findings suggest that while theoretical content is being effectively delivered, opportunities for hands-on application are limited. Nearly half of students (47.6%) believed no changes were needed. Others supported curriculum enhancement, including more AAW topics (12.5%) and a larger practical component (12.8%). Smaller proportions recommended specific improvements (5.59%) and more in-depth information (3.72%). A notable portion (6.91%) gave no comment due to lack of prior exposure, while 8.51% cited “other” reasons or “did not know” (2.39%). Preferences varied across institutions, with a general emphasis placed on experiential learning and theoretical-practical alignment (Table 3).

Despite only 19.2% of institutions including AAW content, nearly half (47.6%) felt no curriculum changes were necessary. This may reflect limited awareness of welfare education needs, as 6.91% of students could not comment due to little prior exposure. Economic factors also appear relevant (69.9% earn <10 million VND monthly), which may lead them to prioritise practical skills over emerging welfare topics. For lower-income students, concern about extending study time and delaying entry into employment may further influence their views on curriculum requirements. These patterns highlight how material and economic circumstances influence student attitudes, even in the presence of their recognition of AAW value.

### 3.3. Educators Perspectives on AAW

Among surveyed educators, satisfaction with the inclusion of AAW in tertiary curricula varied. At institutions where AAW was included, 23.4% reported its inclusion across various aquaculture-related subjects, rather than offered as a standalone course. Regarding satisfaction with how AAW was included in the curriculum, 27.7% of educators were unsure, and 31.9% were unsatisfied. Only a small proportion (4.26%) expressed satisfaction with AAW content across all programmes, while 36.2% were satisfied with its inclusion limited to certain programs.

Some educators (19.2%) confirmed that AAW was already included in their institution’s curriculum; 46.8% reported that inclusion was planned but not yet implemented; 19.2% were unsure and 14.9% reported that AAW was excluded, with no plans for future integration. This suggests that the education system is in a transitional phase. However, the 14.9% remain resistant or unprepared for this inclusion, indicating ongoing barriers within parts of the system. Nearly half (48.9%) either did not respond or provided invalid responses, pointing to potential gaps in knowledge or engagement with curriculum development. Concerning future integration, 51.1% recommended incorporating AAW into existing modules, while only 2.13% suggested establishing a new subject. A notable proportion did not provide specific answers (19.2%), were unsure (27.7%), or could not specify changes due to the current exclusion of AAW in their curricula (6.38%). Educators proposed several strategies for improving AAW instruction, the most frequent recommendation (27.7%) being the development of dedicated curriculum content (Table 4).

Anticipated challenges to curriculum reform were also identified. The most frequently cited barriers being related to curriculum structure, educational alignment (21.3%), and to limited awareness or understanding of AAW (19.2%) (Table 5).

Most educators with more than 15 years of experience (63.8%), were trained in production-focused systems and may have had limited exposure to animal welfare frameworks. This can make incorporating welfare content challenging. Their preference for integrating welfare content into existing programmes (51.1%), rather than creating dedicated courses (2.13%), reflects the awareness of institutional constraints and the production-oriented nature of the current system.

### 3.4. Aquaculture Sector Stakeholders’ Perspective on AAW

Stakeholders (70.6%) reported that graduates were able to identify AAW issues; however, the basis for this confidence was unclear for 41.2% of respondents. With few institutions currently including AAW content, and only a minority reporting confidence in graduates’ welfare skills, the findings suggest that AAW is still a developing area within Vietnamese aquaculture training. Among those who provided reasons, confidence was attributed to various factors: (1) observations of employees during their duties; (2) selection of aquatic feed by employees; (3) verbal interactions and information exchanges; (4) review of curriculum vitae during recruitment; (5) annual examinations; (6) employees’ seafood consumption choices; (7) adherence to company animal welfare protocols; (8) periodic evaluations of employees’ handling of AAW issues; and (9) training gained through academic programmes, company-led initiatives, or self-study.

Stakeholder groups identified several key areas where graduates could contribute greater value to the aquaculture industry. Aquaculture emerged as the most frequently cited area (21.4%), reflecting the sector’s strong demand for skilled personnel in production and technological applications. Other notable areas included welfare research (17.6%), policy and legislation development (12.2%), industry-relevant practices (13.7%), and conservation (16.0%), indicating growing attention to sustainability and ethical practices in aquatic resource management. Public awareness and advocacy (15.3%) were also emphasised, suggesting a need for improved communication and greater stakeholder engagement. Lower response frequencies for industry practices (13.7%) and policy and legislation development (12.2%) may reflect existing capacities or a lower perceived need for improvement. These results highlight the importance of aligning academic programmes with practical aquaculture competencies while integrating cross-cutting themes such as welfare, conservation, and public engagement to enhance graduate preparedness and sectoral impact. In terms of support offered by stakeholders to enhance student learning in AAW, paid internships (0–6 months) received the highest level of support (32.6%). This was followed by short-term practical exposure integrated into elective modules (28.3%). Stakeholders recognised the value of practical demonstrations by academics and supported the use of their facilities for this purpose (19.6%). In contrast, less than 19.6% of stakeholders supported the use of their facilities for research (15.2%) or unpaid internships (4.35%), indicating a strong commitment to hands-on, experiential training.

This aligns with students’ requests for more practical learning opportunities, showing a preference for hands-on experience alongside theoretical instruction. Additionally, support for using facilities for academic demonstration (19.6%) and student-led research (15.2%) signals opportunities for academic-industry collaboration. Unpaid internships were the least supported (4.35%), possibly reflecting concerns about equity and effectiveness. Stakeholder responses indicate an understanding that building AAW capacity involves structured investment and support rather than relying solely on voluntary effort. This highlights the need for dedicated resources, formal training programmes, and partnerships to develop welfare standards and integrate this within curricula. These findings emphasise the need to promote structured, practice-oriented learning experiences that foster both academic and professional development in the aquaculture and aquatic welfare domains.

### 3.5. Understanding of AAW Among Survey Respondents

Respondents’ understanding of AAW welfare varied across the surveyed groups. While 53.8% of students reported familiarity with the concept of the Five Freedoms of Animal Welfare, only 5.57% accurately listed all five. The contrast between the 37.1% of students who reported being “very satisfied” with the learning resources and the limited internalisation of core concepts reflects an awareness-depth gap. This gap likely results from factors such as superficial exposure to welfare topics in existing courses, limited practical experiences, and the absence of structured, dedicated AAW modules. The traditional production-focused approach in Vietnamese aquaculture education may reinforce this gap by prioritising technical production skills over welfare.

Among the three groups, stakeholders had the lowest conceptual understanding (21.3%), followed by students (29.4%) and educators (32.5%). Standardised perception scores (on a 100-point scale) were highest among stakeholders (73.3%), slightly ahead of students (69.2%) and educators (68.9%) (Table 6). 

Few respondents recognised the role of welfare in different aspects of animal use, such as farming, transport, and handling (14.8% among educators, 7.52% among students, and 17.2% among stakeholders) (Table 7). 

**Figure 4:**
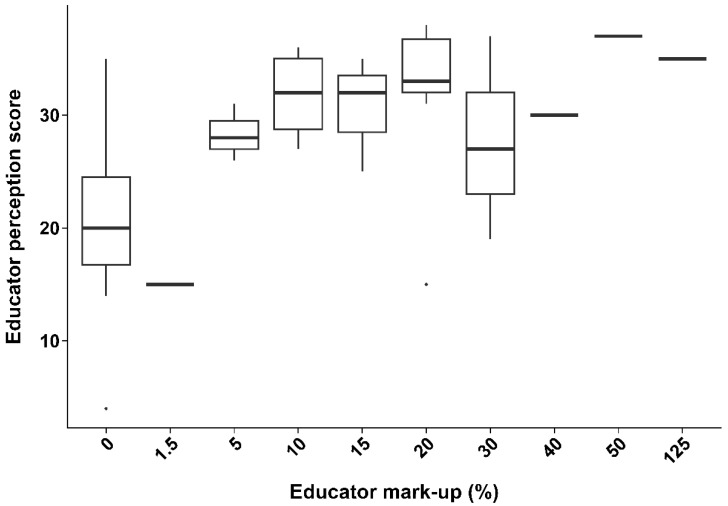
Illustration of the association between perception score and mark-up (%) from educators (n = 47) in Vietnamese tertiary institutions surveyed for the perception study (*p* < 3.18 × 10^−7^, df = 45, Kendall–Theil–Sen). Boxplots show the median, interquartile range, and range of perception scores, with outliers represented by dots.

**Figure 5:**
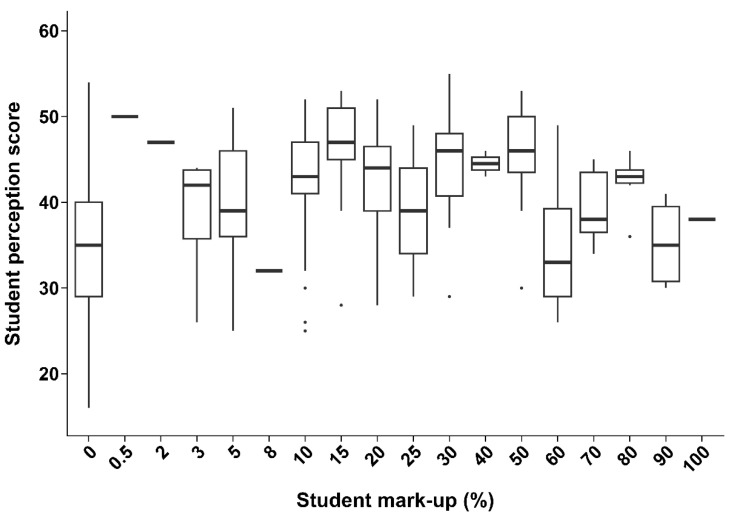
Illustration of association between perception score and mark-up (%) from students (n = 359) in Vietnamese tertiary institutions surveyed for the perception study (*p* < 2 × 10^−16^, df = 357, Kendall–Theil–Sen). Boxplots show the median, interquartile range, and range of perception scores, with outliers represented by dots.

Regarding consumer behaviour, the most accepted premium for higher-welfare aquatic products fell within the 6–10% range. Significant positive correlations (*p* < 0.05) were observed between perception scores and WTP for both educators and students, as illustrated in Figure 4 and Figure 5.

Students’ median perception scores rose from approximately 30 at a 0% mark-up to the high 30 s to mid-40 s at a 5–15% mark-up. However, beyond the 30% premium, perception scores showed no clear trend, indicating a weaker correlation between WTP and AAW perception at higher prices. The observed non-linear relationship highlights that perception alone does not fully explain the WTP. This positive correlation between perception and WTP premiums suggests that improving consumer education on AAW standards could encourage market-driven demand. Economic incentives have the potential to support and reinforce animal welfare goals. Given that 12.2% of stakeholders identified policy development as a priority, the role of consumer education may be underappreciated.

**Figure 6:**
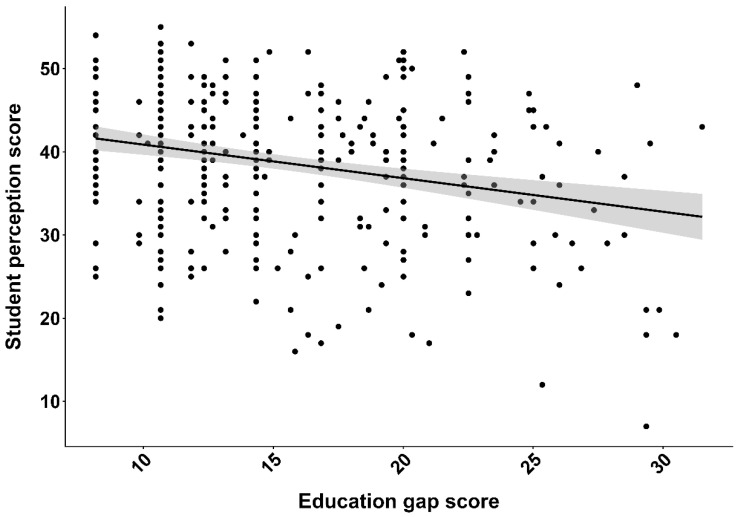
Illustration of the negative relationship (slope −0.28) between student (n = 359) perception scores and their education gap scores (*p* < 1.25 × 10^−12^, df = 357, Kendall– Theil– Sen), suggesting that students with larger gaps in aquatic animal welfare have associated lower perceptions. The shaded region represents the 95% confidence interval around the Kendall–Theil–Sen fitted trend.

Additionally, a small but statistically significant inverse relationship was observed between students’ perception scores and their education gap scores (slope = −0.28; *p* = 1.25 × 10^−12^) (Figure 6). However, no significant differences in education gap scores or perceptions scores were detected across institutions, majors, or academic years (*p* > 0.05), suggesting a consistent pattern of understanding among students regardless of disciplinary background or academic progression. This uniformity likely reflects the limited integration of structured AAW content within existing curricula across programmes. Most students (74.9%) exhibited low education gap scores, while only a small fraction (0.56%) were classified as having high education gaps. The mean education gap score was 15.3 ± 5.4, and most students fell within the moderate range (11–20). The observed inverse relationship between perception and education gap scores suggests that students with stronger foundational knowledge have fewer knowledge gaps in AAW. This pattern highlights differences in knowledge amongst students and may help identify weaknesses and areas for improvement in teaching content and methods.

The scatterplot shows a general trend as education gap scores increase, indicating greater perceived deficiencies in AAW instruction, and student perception scores decrease. Although this inverse relationship links perceived curricular gaps with weaker welfare perceptions, the modest effect size suggests that other influences, such as informal learning, cultural background, and personal values, also substantially shape attitudes.

## 4. Discussion

### 4.1. Knowledge and Awareness Insights on AAW

This exploratory study provides the first empirical evidence on perceptions of AAW and related educational gaps among students, educators, and key stakeholders in South and South-Central Vietnam. While several Asian studies have examined the perceptions of either tertiary students or aquaculture industry stakeholders on specific aspects of aquatic animal welfare, or animal welfare more broadly, there are currently no national studies in Asia that employ a three-group design that simultaneously surveys students, educators, and industry stakeholders in AAW with an aquaculture focus. This makes the present study the first of its kind in both national and regional contexts. Given that Vietnam is among the world’s top five aquaculture-producing countries, the insights generated from this research have significance not only for national curriculum development but also for international discussions on AAW education and governance across major aquaculture-producing regions. In comparison to emerging animal welfare studies from other countries, including those in Asia [13], similar patterns of high general awareness with limited deep-level understanding of welfare principles were observed.

When viewed through the Knowledge-Attitude-Practice (KAP) model, the survey results reveal a nuanced picture of how students, educators, and stakeholders understand and engage with AAW. In the context of this study, the application of the KAP model evaluates the knowledge and awareness of survey participants, their beliefs and feelings (attitudes), and their resultant behaviours concerning AAW. It helps us understand whether their awareness and attitudes towards AAW translate into meaningful behaviour [14].

**Figure 7:**
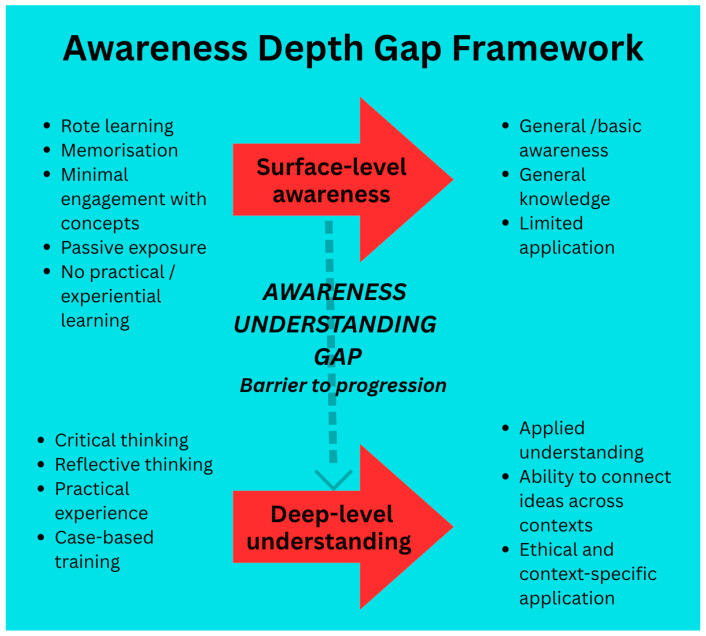
The Awareness Depth Gap Framework illustrates how surface-level awareness in AAW fails to progress into deeper conceptual understanding or applied competence without structured, experiential, and reflective learning processes. The broader learning and institutional environment (*blue*) shows critical learning states indicated by the arrows, contrasting surface-level awareness and deep-level understanding *(red)*. The persistent but bridgeable gap between surface level awareness and meaningful understanding is highlighted by the dashed pathway *(grey)*.

During examination of the knowledge dimension, while awareness of key welfare principles, such as the Five Freedoms of Animal Welfare, was broadly recognised, understanding of details was limited. Most students scored “high” to “very high” (87.8%) on perception scores (Table 6), indicating general awareness of AAW, yet they still lacked deeper conceptual understanding. While 53.8% of students reported recognising most or all components of the Five Freedoms of Animal Welfare, only a small fraction (5.57%) could accurately articulate them. Furthermore, 17.0% of students demonstrated a lack of conceptual understanding of animal welfare principles. Despite reporting moderate to high satisfaction with teaching quality and resources, only 17.6% of students felt confident discussing AAW topics (Figure 3), highlighting a pattern of awareness without corresponding competence. Fragmented AAW coverage (19.2% of institutions) and limited hands-on experience together reinforce the gap between knowing welfare principles and applying them in practice. These patterns illustrate an awareness-depth gap, with surface recognition of welfare principles not matched by a deeper understanding required for ethical competent practice. This “awareness-depth gap” reflects how cultural, societal, and educational factors often lead to surface-level knowledge, without developing critical thinking, integration, or practical skills. It aligns with previous studies suggesting that positive perceptions of animal welfare do not necessarily translate into applied knowledge or ethical decision-making capabilities [15,16,17]. Biggs and Tang [18] distinguish between surface and deep approaches to learning. In surface learning, students focus on memorisation and meet basic requirements, while deep learning involves understanding underlying principles, connecting ideas across contexts, and applying them in new situations. We propose the Awareness Depth Gap Framework (Figure 7), which illustrates how surface-level awareness observed in this study does not progress into deeper conceptual or applied competence without structure, experiential, and reflective learning. This Framework offers a novel way to pinpoint where students get “stuck” at surface-level awareness, and to guide targeted curriculum interventions that encourage deeper learning. It represents a novel conceptual tool for AAW education research, as previous studies have identified gaps between attitudes and practice, but few have provided a structured explanation for how and why learners remain at a superficial level of understanding.

The limited statistical variation in education gap scores across majors, years, and institutions suggests the shortfall in structured AAW education, further supported by the uncertainty of educators regarding curriculum coverage. The limited awareness of legal obligations related to AAW, especially among stakeholders (3.45%), reflects the absence of comprehensive AAW laws in Vietnam [4,19].

The statistically significant negative association between student perception scores and education gap scores (Kendall–Theil–Sen slope = −0.28, *p* < 1.25 × 10^−12^) (Figure 6) highlights the positive role formal education plays in awareness, ethics, and welfare attitudes. These findings align with previous studies showing that educational exposure strongly influences animal welfare perceptions [20]. The variation around the trend line shows that, although curriculum depth has a statistically significant effect on student perceptions as reported by Mijares, Sullivan [20], Sinclair, Lee [13], informal learning, cultural background, and personal values also shape student attitudes toward animal welfare. This highlights the need for a comprehensive, multi-dimensional approach that combines formal instruction with experiential learning. These findings align with prior research highlighting the multi-dimensional nature of welfare perception development [21,22,23].

Among educators, the high rate of unclear (48.9%) or noncommittal responses (46.8%) concerning the inclusion of AAW in the curriculum likely reflects a limited awareness of curriculum content, poor communication regarding curricular updates, or low institutional prioritisation of welfare topics. Although 70.6% of stakeholders believed graduates were trained sufficiently in AAW, 41.2% could not cite examples to support this view. This discrepancy illustrates the classic knowledge–action gap, where awareness does not always translate into behaviour or applied skills [24].

### 4.2. Attitude Insights on AAW

Cultural and societal values in Vietnam often foster only superficial awareness of animal welfare, which may not translate into genuine concern or motivation to apply humane and ethical practices [25]. Shaping positive attitudes toward animal welfare requires not merely awareness but engagement that encourages reflection, critical thinking, and ethical reasoning. Regarding attitudes, the high perception scores of students (87.8%), stakeholders (94.1%), and educators (80.9%) indicate a strong value placed on AAW within Vietnamese aquaculture education, even in the context of existing curricular limitations. This positive attitude provides a solid foundation for fostering improved welfare practices, since individuals who view AAW as important are more likely to apply this to real-world contexts with the right guidance and support. Most students (89.7%) recognised that education in AAW could enhance their future career opportunities, reflecting an awareness of its relevance within the aquaculture sector. This perception aligns with global trends that recognise animal welfare as an important part of sustainable aquaculture, particularly in countries involved in international trade and export [26].

However, despite satisfactory ratings on teaching quality (79.9%) and learning resources (77.7%), only 17.6% of students felt confident discussing AAW topics. This not only highlights a gap between perceived adequacy and preparedness but also limited exposure to AAW content and practical experience. These findings correspond with the low education gap scores reported by most students (74.9%), reflecting moderate satisfaction and awareness. Students’ preference for expert-led workshops and hands-on experiences suggests that, although AAW awareness is increasing, many find the current curriculum lacking in practical depth. The lack of statistically significant differences in perception scores among educators with different qualifications suggests that academic level alone may not influence welfare-related perceptions. Instead, previous research indicates that individual factors, such as empathy, ethical orientation, and personal values, may influence how educators engage with and prioritise welfare content [26,27]. These intrinsic factors can determine whether educators advocate for AAW inclusion, even in the absence of strong policy directives. Therefore, fostering awareness and ethical commitment among educators may be as important as policy reform in strengthening AAW education in Vietnam’s aquaculture sector [28,29]. The high proportion (48.9%) of uncertain or incomplete responses among educators may reflect knowledge gaps and varying attitudes toward AAW education. This includes the perceived importance of welfare content and willingness to engage in curriculum development. Therefore, faculty support is essential for successful welfare education reform [13,21].

In contrast with Western studies, which often identify gender and age as key predictors of animal welfare attitudes [9,27]. This study found no significant effects (*p* > 0.05) on perception scores, despite balanced gender representation among students and educators. This suggests that Vietnam’s cultural and educational uniformity, with centralised curricula and relatively homogeneous socio-cultural norms, may reduce these influences on attitudes towards AAW. Similarly, household income showed no significant effect on attitudes because of social desirability bias, where respondents align their answers with perceived social norms [30]. This aligns with Southeast Asian research, where shared cultural values and standardised state-led curricula limit individual differences in attitudes toward sustainability and welfare [31,32]. These results suggest that while demographic predictors explain welfare attitudes in Western contexts, their influence appears weaker in Vietnam, where structural and cultural factors are more important.

Higher AAW perception scores among both educators (Figure 4) and students (Figure 5) were positively associated with a greater WTP premium for higher-welfare aquaculture products. This suggests the recognition that higher welfare standards justify increased consumer costs and reflect how knowledge can shape ethical purchasing decisions. Greater welfare awareness and understanding can enhance consumers’ willingness to support ethical production through market behaviour, which is a trend also seen in other studies linking welfare knowledge to WTP [27,33,34]. Notably, the most accepted price premium among respondents was 6–10%, supporting research that shows moderate mark-ups being the most acceptable for welfare-certified products [35,36]. In contrast, stakeholders’ perception scores showed no statistically significant association with WTP, possibly reflecting that practical or economic constraints outweigh welfare considerations in industry contexts.

### 4.3. Behavioural Practices Related to AAW

To fully understand the implications of rising awareness of AAW in Vietnam, it is crucial to examine how current knowledge and attitudes translate into concrete actions, and how evolving perceptions may shape future welfare practices as educational and institutional reforms continue to advance.

The high seafood consumption rates among educators and stakeholders with strong AAW perceptions suggest that positive attitudes do not always translate into practice, likely influenced by cultural, dietary, or economic factors. This is consistent with the cognitive dissonance theory [37] and highlights the need for practical training and interventions that support individuals in translating their ethical attitudes into practice. This may include informed decision-making, welfare-conscious product choices, or applied welfare improvements in aquaculture operations. The 6–10% price premium offers a practical benchmark in Vietnam for implementing higher-welfare aquaculture practices. It highlights an opportunity to translate consumer awareness into actionable strategies, including marketing welfare-certified products, engaging communities, and integrating welfare considerations into policy and public outreach. Linking WTP to real-world applications illustrates how knowledge and attitudes can shape practical decision-making across the aquaculture sector.

The low recognition of welfare considerations across farming, transport, and handling (14.8% among educators, 7.52% among students, and 17.2% among stakeholders) suggests that respondents have few opportunities to translate theoretical knowledge into practical skills. This reinforces the need for alternative strategies such as case-based learning, collaborative group discussions, and experiential training aimed at bridging the gap between theoretical awareness and practical application [18,38].

The Five Freedoms of Animal Welfare, cited most frequently by students (53.8%) (Table 7), provide a familiar and accessible foundation for integrating practical AAW teaching into aquaculture programmes. Students prefer expert-led workshops and practical experience, whilst most aquaculture stakeholders (88.2%) expressed support for curriculum reform and more practical training opportunities. These findings highlight the value of a mixed-delivery approach that blends welfare topics into core courses, expands field-based training, and includes expert-led sessions. As suggested by Deemer and Lobao [39], integrating real-world case studies, hands-on husbandry practices, and critical thinking exercises can help bridge the gap between normative attitudes and functional knowledge. The lack of statistical variation across years and majors further emphasises the need for a stepwise curriculum approach that gradually increases experiential learning as students progress in their studies. More structured, sector-wide integration of AAW content into formal aquaculture curricula across programmes could help close gaps demonstrated by education gap scores. Aligning AAW principles with professional development goals could boost student motivation, build confidence, and enhance the long-term impact of welfare education in this group.

Educators reported several systemic barriers to integrating AAW into aquaculture curricula, including overloaded syllabi, misalignment with MOET frameworks, limited expertise, and limited institutional or stakeholder support (Table 5). These challenges contribute to the inconsistent or minimal integration of welfare content in everyday teaching practice and maintain the awareness-depth gap. Effective curriculum reform and improved welfare practice in aquaculture require not only curriculum redesign, but also faculty development, institutional commitment, targeted funding, and engagement from industry and policy stakeholders. As seen in many low- and middle-income countries, where economic pressures and utilitarian views of animals often shape welfare priorities, the need for stronger enabling structures cannot be overstated. Effective AAW implementation also requires coordinated policy frameworks, clear institutional mandates, and capacity-building initiatives that equip educators to deliver welfare content consistently and meaningfully.

Stakeholders prioritise technical skills (21.4%), welfare-related research (17.6%), and conservation (16.0%), reflecting demand for graduates skilled in theoretical knowledge with practical AAW skills. Bridging the gap between academic training and industry needs requires joint curriculum committees, structured internships, and regular curriculum reviews. As Parajuli, Pham [40], Bui and Takuro [41] highlighted, limited university and industry collaboration in Vietnam arises from weak private-sector engagement and limited focus on industry-relevant research.

Finally, regardless of how AAW content is delivered, reforms must also address the access barriers faced by lower-income students in Vietnam. As noted by Parajuli, Pham [40], financial barriers can significantly limit fair access to higher education, particularly in technical fields. Therefore, targeted scholarships, flexible delivery models, and inclusive teaching are key to improving access to welfare education and its impact on the aquaculture industry. These behavioural patterns underscore the need for structured curriculum reform, as highlighted in the Awareness Depth Gap Framework. By supporting deeper, more engaged learning, students progress from basic awareness to confident, competent welfare practices in real aquaculture settings.

Collectively, these findings highlight clear entry points for aligning Vietnam’s aquaculture education and practice with WOAH aquatic animal health and welfare standards. The patterns observed, particularly the uneven integration of AAW content, uncertainty about curriculum coverage, and limited awareness of regulatory obligations, indicate where regional guidance and harmonised minimum standards that reflect WOAH principles could be valuable. Strengthening links between tertiary education and industry needs through structured practical exposure, internships, and shared training facilities is also consistent with WOAH’s emphasis on multi-sector collaboration (WOAH, 2025) [5]. Vietnam’s welfare education gaps must be viewed within a broader policy context. As a WOAH Member Country, it has access to training, technical support, and legislative guidance that support the development of national animal welfare governance (WOAH, 2025) [5]. The Asia-Pacific Regional Animal Welfare Strategy (RAWS), established in 2008, offers a shared framework that helps countries align welfare initiatives with regional priorities (WOAH RR-Asia, 2025) [5]. Strengthening AAW education, therefore, complements ongoing WOAH efforts to harmonise welfare standards across sectors and regions. This positions Vietnam to implement welfare improvements in line with internationally recognised frameworks.

### 4.4. Potential Sources of Bias

Several types of response bias may have influenced the findings. Participants may have been affected by social desirability bias, choosing answers they felt were more acceptable rather than reflecting their genuine views or behaviours. A knowledge action gap may also be present, since what people say they believe does not always match what they do in practice. Cognitive dissonance could have played a role if respondents adjusted their answers to fit what they felt they should value. Cultural and contextual factors may also have shaped responses. Participants from different regions, educational settings, or linguistic backgrounds within Vietnam might have interpreted certain questions differently, creating cultural sensitivity bias. Conducting surveys in person may have introduced additional bias through misunderstanding, fatigue, or subtle interviewer influence. The education-gap metric relied on self-reported information, meaning that some respondents may have over- or under-estimated their own knowledge or abilities. Differences in how AAW is taught across institutions could also have contributed to curriculum delivery bias, since participants had varying levels of exposure to the topic. Finally, institutional commitment bias may have arisen if educators were uncertain about, or lacked support for, implementing curriculum changes.

## 5. Conclusions

This study employed a mixed-methods survey design incorporating the Awareness Depth Gap Framework to assess how education influences perceptions of AAW. While the findings provide valuable insights, limitations related to bias and sampling highlight the need for methodological refinements in future research. These include using randomised, stratified sampling of stakeholders to ensure diversity, and validating perception measures through practical competency assessments.

This study shows an association between the education gap and AAW perceptions, but causal links remain unverified, and longitudinal or experimental studies are needed to evaluate the impact of educational interventions. Future work should pilot targeted AAW training and explore additional influences on perceptions, such as informal learning, peer networks, and institutional culture. Replicating this study in other aquaculture-producing nations would help evaluate the broader applicability of these findings. A comparative analysis across developing aquaculture countries could identify Vietnam’s relative progress and inform best practices for regional AAW education.

Given the inconsistent delivery of AAW content and varying institutional commitment, curriculum reform is essential. Standardised, in-depth AAW training integrated across aquaculture programs is needed to address both the awareness depth gap and knowledge-action gap. Experimental or longitudinal designs can rigorously evaluate the effectiveness of new training modules and capacity-building initiatives for educators and stakeholders. Future work could also focus on developing a practical framework for implementing AAW education. This could combine ethics, legislation, and practical training to support consistent adoption by institutions and policymakers. Policy support is crucial to institutionalise these reforms. Priorities include mandating standardised AAW curricula, strengthening educator training, promoting university-industry collaboration to improve practical exposure, and increasing access for underrepresented groups. Adopting community development approaches that link animal welfare improvements with human well-being will ensure ethically sound and socially sustainable outcomes.

## Figures and Tables

**Table 1:** Scoring scale for education gap scores for the student survey (n = 359) that were calculated based on four criteria: (i) confidence in discussing AAW topics, (ii) perception of curriculum sufficiency in covering AAW, (iii) satisfaction with materials, resources, and overall learning experiences, and (iv) satisfaction with instructors teaching AAW.

Education Gap Levels	Score Range	Description
Very low	0–10	Students who have a high level of confidence concerning AAW knowledge are satisfied with their curriculum and instructors, and feel they are taught adequate AAW.
Low	11–20	Students who are moderately confident, relatively satisfied with learning materials and instructors, but may feel uncertain about the sufficiency of AAW coverage in the curriculum.
Moderate	21–30	Students who have low confidence are dissatisfied with learning resources or instructor quality and perceive a lack of sufficient AAW education.
High	31–40	Students who lack confidence, express dissatisfaction or disinterest in AAW content, and feel the curriculum does not adequately address AAW.

**Table 2:** Demographic details included age, religion, income, and experiences of survey participants (i.e., students (n = 359), educators (n = 47), and stakeholders (n = 34)).

Variables	Category	Students (n = 359)	Educators (n = 47)	Stakeholders (n = 34)
Gender (%)	Male	55.2		
	Female	43.9		
	Undisclosed	0.9		
Age (Year) (%)	18–21	61.6	-	-
	22–25	37.3	-	-
	26–30	0.6	-	-
	>30	0.6	-	-
	36–45	-	66.0	55.9
	46–55	-	29.8	11.8
	56–65	-	4.3	8.8
	25–35	-	-	23.5
Religion (%)	Buddhism	24.0	8.5	8.8
	Catholic	4.2	4.3	5.9
	None	66.0	76.6	70.6
	Other	5.8	4.3	8.8
	No answer	-	6.4	5.9
Monthly income (Million VND)	<10	69.9	10.6	17.6
	10–20	22.8	61.7	32.4
	20–30	5.3	17.0	23.5
	>30	2.0	10.6	20.6
	No answer	-	-	5.9
Teaching experience (%)	0–5 years	-	4.3	-
	6–10 years	-	10.6	-
	11–15 years	-	21.3	-
	>15 years	-	63.8	-

**Table 3:** Number of Vietnamese tertiary students (n = 359) recommending various methods for including and improving AAW content in the curriculum, across the different institutions sampled.

Tertiary Institution	A New Subject	Integration into Technical Subjects	More Practical Experience	Talks, Seminars, Workshops with Specialists
Bạc Liêu College of Economics and Technology (n = 7)	2	2	3	0
Bạc Liêu University (n = 34)	19	19	17	14
Cà Mau Community College (n = 7)	3	2	1	0
Cần Thơ University (n = 150)	73	76	94	66
Đồng Tháp Community College (n = 4)	1	2	1	1
Ho Chi Minh City University of Agriculture and Forestry (n = 52)	15	35	25	16
Nha Trang University (n = 41)	14	19	17	21
Sóc Trăng Vocational College (n = 11)	4	8	8	6
Southern Agricultural College (n = 2)	1	0	0	0
TràVinh University (n = 51)	17	16	25	23

**Table 4:** Collective recommendations by educators (n = 47) for improving the teaching of AAW at their institutions and departments in South and South-Central Vietnam.

Recommendation Category	Frequency (%)	Key Suggestions
Dedicated curriculum content	27.7	Introduce dedicated modules or subjects, integrate into aquaculture theory, or offer elective courses.
Awareness and ethical understanding	10.6	Enhance knowledge through the KAP (Knowledge, Attitudes, and Practices) model, ethical principles, and welfare awareness.
Practical welfare applications	10.6	Include topics such as anesthetic use, sustainable husbandry, humane harvesting, and environmental management.
Interdisciplinary and sector-specific content	12.8	Align AAW with fisheries law, aquatic processing, scientific research, and sector-specific requirements.
Workshops and curriculum updates	6.38	Organize workshops with expert input and ensure regular updates on regulations and standards.
Resource development and expertise	4.26	Develop educator expertise and allocate resources for effective AAW teaching.

**Table 5:** Anticipated challenges to AAW curriculum integration as reported by educators (n = 47).

Category	Challenges	Response Frequency (%)
Awareness and understanding of AAW	(i) Lack of understanding and awareness of AAW’s importance across various societal groups, including cultural contexts. (ii) Limited familiarity with recent AAW developments, particularly among farmers and consumers. (iii) Prevailing attitudes that prioritise aquatic animals’ utility over welfare considerations.	19.2
Curriculum and educational structure	(i) Curriculum overload and limited time or resources. (ii) Need for alignment with the Ministry of Education policies and frameworks. (iii) Fragmented AAW coverage across multiple modules and absence of a dedicated subject.	21.3
Economic and institutional constraints	(i) Emphasis on economic efficiency and profit margins in the industry at the expense of welfare considerations. (ii) High financial investment required to incorporate AAW into curricula.	10.6
Expertise and training	(i) Lack of field-specific AAW expertise and insufficient educator training. (ii) Reluctance among educators to invest in skill development. (iii) Absence of a national or institutional reference model for AAW.	14.9
Resistance to change and culture	(i) Institutional resistance because of entrenched traditions and low levels of interest in AAW among some individuals or departments.	4.00
Information and resources	(i) Inadequate access to AAW-related information and teaching resources. (ii) Lack of established national legislation governing animal welfare.	3.00
Uncertainty	(i) Ambiguity surrounding the potential outcomes of curriculum changes involving AAW.	8.00

**Table 6:** Score scale for perception scores (standardized to 100), score ranges, and scores for students (n = 359), educators (n = 47), and stakeholders (n = 34) in South and South-Central Vietnam, for welfare scoring questions from the perception survey (% of responses).

Respondent Category	Low	Moderate	High	Very High
	0–25	26–50	51–75	76–100
Students (n = 359)	0.56	11.7	48.8	39.0
Educators (n = 47)	2.13	17.0	31.9	48.9
Aquaculture sector stakeholders (n = 34)	0	5.88	52.9	41.2

**Table 7:** Responses from Vietnamese students (n = 359), educators (n = 47), and stakeholders (n = 34) (%) on their understanding of AAW. Figures reflect the percentage of welfare criteria mentioned per response, not individual respondents, which are based on the total criteria identified within each respondent category.

Respondent Category	Human Concern (Ethics, Morals, Humane Treatment) (%)	Legal Responsibility (Regulations & Rights) (%)	5 Freedoms of Animal Welfare (%)	Impact on Variety Activities (Farming, Harvest, Slaughter, Transport, Handling, etc.) (%)
Students (n = 359)	32.6	23.7	53.8	7.52
Educators (n = 47)	29.5	27.9	27.9	14.8
Aquaculture sector stakeholders (n = 34)	44.8	3.45	34.5	17.2

## Data Availability

Data are available from the corresponding author on request.

## Supplementary Materials

The following supporting information can be downloaded at: https://www.mdpi.com/article/10.3390/ani16010026/s1; Table S1: Summary of tertiary institutions sampled in South and South-Central Vietnam; Table S2: Survey questions for the perception study on educators within South and South-Central Vietnam; Table S3: Survey questions for the perception study on students within South and South-Central Vietnam; Table S4: Survey questions for the perception study on industry stakeholders within South and South-Central Vietnam; Table S5: Summary of scoring questions from the perception survey; Table S6: Summary of questions for the education gap score. References [42,43] are cited in the Supplementary Materials.
