# Supplementary material for: Assessing Perceptions Toward Aquatic Animal Welfare: A Study on the Perspectives of Educators, Students and Aquaculture Industry Stakeholders in South and South-Central Vietnam
Source: Animals (Basel). 2025 Dec 22;16(1):26. doi: 10.3390/ani16010026 (PMC12784893; doi:10.3390/ani16010026)
Supplement: Supplementary file 1 [file animals-16-00026-s001.zip › animals-3952053-supplementary.pdf]

I. Supplementary methods

S1. Survey instrument validation and pilot study:

Questionnaires were validated by five TVU members using face and content validation methods [42]. A pilot study with seven participants (students, educators, and stakeholders) assessed clarity, translation accuracy, and cultural alignment. Revisions to the survey instrument were made accordingly. Internal reliability, measured using McDonald's omega in RStudio, showed acceptable values: 0.71 (students), 0.70 (educators), and 0.84 (stakeholders) [43].

S2. Inclusion criteria for sampling:

All colleges with eligible student enrolments were included, while (randomly sampled) universities were required to have a minimum of 100 students enrolled (in total) in relevant programmes to ensure adequate sampling frames. Educators (n=47) were randomly selected based on institutional ratios (one educator per 50 students at universities and one per 20 students at colleges) and involvement in aquatic animal-related curricula

S3. The variables investigated in this study included gender, age, religion, nationality, household income, views on Vietnamese culture, and seafood consumption, which were collected across all respondent groups. In addition, specific variables were considered for each group. For educators, these included their professional rank, highest qualification, and years of teaching experience. For students, these include major, academic year, other tertiary education background, and intended future occupation. No additional variable were collected for aquaculture sector stakeholders beyond those common across groups.

S4. Perception scoring questions:

Students answered seven questions assessing their perceived importance of animal welfare in Vietnamese culture; factors contributing to good AAW; welfare considerations in aquaculture; WTP for higher-welfare products and acceptable percentage mark-ups they would accept; and relevance of AAW education to their studies and careers. Educators and stakeholders answered the same first five questions as students. Educators additionally rated the perceived importance of AAW inclusion for learners in aquaculture and animal health professions. Stakeholders assessed its relevance to the industry for students and their willingness to support tertiary institutions in enhancing learners' experience.

2. Supplementary Tables:

Table S1. Summary of tertiary institutions sampled in South and South-Central Vietnam, including majors of interest, and student (n=359) and educator (n-47) sample sizes. Where multiple majors were present at an institution, sample numbers are indicated in brackets.

Institution names	Majors	Students	Educators
**Colleges**		**31**	**9**
Bạc Liêu College of Economics and Technology	- Aquaculture diploma	7	2
Cà Mau Community College	- Aquaculture diploma	7	2
Đồng Tháp Community College	- Aquaculture diploma	4	1
Sóc Trăng Vocational College	- Aquaculture diploma<br>- Engineer aquaculture post- harvest	11	3
Southern Agricultural College	- Aquaculture diploma	2	1
**Universities**		**328**	**38**
Bạc Liêu University	- Engineer aquaculture	34	4
Cần Thơ University	- Engineer aquatic resource management (n=17)<br>- Engineer aquaculture (n=76)<br>- Engineer aquaculture pathology (n=22)<br>- Engineer aquaculture post-harvest (n=35)	150	15
Ho Chi Minh City University of Agriculture and Forestry	- Engineer aquatic resource management (n=7)<br>- Engineer aquaculture (n=19)<br>- Engineer aquaculture pathology (n=6)<br>- Engineer aquaculture post-harvest (n=20)	52	5
Nha Trang University	- Engineer aquaculture	41	6
TràVinh University	- Engineer aquaculture	51	8

Table S2: Survey questions for the perception study on educators within South and South-Central Vietnam. Questions in bold were compulsory. Where options are provided for certain questions, these are highlighted in the options column. Questions shaded in grey are welfare scoring questions.

No.	Questions	Options
**1-3**	Full name; email address; phone number	
**4**	Please indicate your gender?	Male/ female/ other/ prefer not to say
**5**	Please indicate your age range?	25-35 / 36-45/ 46-55/ 56-65/ >65
**6**	What religion do you identify with?	Buddhism/ Taoism/ Christian/ Atheist or agnostic/ other
**7**	What nationality are you?	
**8**	How much is the monthly household income of the head of your household in VND (Vietnamese Dong)? (this may be yourself or another person)	<30 000 000/ 20 000 000-30 000 000/ 10 000 000-20 000 000/ <10 000 000
**9**	What is the name of the school and department you work for?	
**10**	What is your role/position as an educator at the tertiary institution?	
**11**	How long have you been teaching at any tertiary institution (i.e. not specific where you are currently teaching)?	0-5 years / 6-10 years / 11-15 years / > 15 years
**12**	(i) Educational background: indicate your highest qualification	Bachelors' degree/ Master's degree/ PhD
**12**	(ii) Educational background: your major of highest qualification?	
**13**	In your own words what does animal welfare mean to you?	
**14**	What is the importance of animal welfare in Vietnamese culture and society according to your understanding?	Animal welfare is not important for any animals in Vietnam/ Animal welfare is not important for aquatic animals in Vietnam, but important for non-aquatic animals/<br>AAW has some importance in Vietnam, but I am unsure of the importance/<br>AAW is extremely important in Vietnam /<br>AAW has mostly not been important previously, but is now increasing in importance in Vietnam/<br>I do not know.
**15**	In your opinion which aspects contribute to good AAW? Tick all that apply.	Adequate space for natural behaviour/<br>Respect of species specific behaviours and instincts;

		Consideration of social needs and interactions with its own kind/<br>Access to good water quality and a suitable habitat/<br>Protection from pain, injury, and disease/<br>Access to veterinary care and medical attention when needed/<br>Proper nutrition and feeding practices/<br>Opportunities for mental stimulation/<br>Reduced stress factors/<br>I do not have enough knowledge to answer/<br>Other
**16**	Where do you think animal welfare is an important feature when farming aquatic animals? Tick all that apply.	Handling / transportation/ harvest/ slaughter/ general husbandry/ I do not have enough knowledge to answer / other
**17**	Do you eat aquatic animals (fish or shellfish) and how often?	No/ daily/ at least once a week/ several times a week/ at least once a month/ yes, not often.
**18**	(i) If you had the choice of buying fresh fish or shellfish from your local supermarket which of the products below, would you choose?	Product A (fresh, locally farmed (intensive) (300g), clear air-tight sealed packaging, ~65 000 VND)/ Product B (the same as product A but which is sourced from a farm practising high welfare standard in their husbandry, harvesting, and slaughter of animals)/ it makes no difference to me, so either product.
**18**	(ii) Would you pay extra for product B?	No/ yes
**18**	(iii) If you answered yes to the previous question, how much more would you pay for product B (in%) and WHY?	
**19**	Does welfare of aquatic animals currently feature in the curricula offered at your institution?	Yes/ not yet, and there is not yet an intention to include it/ not yet, but the intention is to include it/ not sure.
**20**	If yes, please describe :<br>(i) how it is included in the curriculum (i.e. stand-alone module or included in another module (name it)),	

	(ii) which majors (name it), and<br>(iii) where possible indicate the total number of credits allocated towards AAW within each major? Example: Major 1, included in 3 modules, 20 credits in total, Major 2, 1 stand-alone module, 15 credits. Please indicate if you do not know.	
**21**	If not yet please suggest how you plan to include the topic of aquatic animal welfare in undergraduate degrees (major, subject, or new subject) at your university? Please indicate if you do not know at this point in time.	
**22**	On a scale of 1-5 how important do you think is the inclusion of AAW in the education of learners in aquaculture and animal health professions?	Score 1-5 (very unimportant – very important)
**23**	How does your institution define 1 credit (i.e. how many hours are allocated tom achieve a study outcome)?	
**24**	Do you think welfare related to aquatic animals is adequately covered in the current curricula offered to undergraduate university students at your institution?	Yes, applicable to all offered programs/ yes, applicable to some offered programs only/ not at all/ I am not sure
**25**	What changes, if any, do you think could be made concerning welfare education on aquatic animals at your institution?	
**26**	Do you expect any challenges in making these changes and what might these be?	

Table S3: Survey questions for the perception study on students within South and South-Central Vietnam. Questions in bold were compulsory. Where options are provided for certain questions, these are highlighted in the options column. Questions shaded in grey are welfare scoring questions.

No.	Questions	Options
**1-3**	Full name; email address; phone number	
**4**	Please indicate your gender?	Male/ female/ other/ prefer not to say
**5**	Please indicate your age range?	25-35 / 36-45/ 46-55/ 56-65/ >65
**6**	What religion do you identify with?	Buddhism/ Taoism/ Christian/ Atheist or agnostic/ other
**7**	What nationality are you?	
**8**	How much is the monthly household income of the head of your household in VND (Vietnamese Dong)? (this may be yourself or another person)	<30 000 000/ 20 000 000-30 000 000/ 10 000 000-20 000 000/ <10 000 000
**9**	Name of the school and department where you are currently registered ?	
**10**	(i) Name of the program (degree/ diploma) you are currently registered in?	
**10**	(ii) What is your major?	
**11**	What academic year are you currently in?	Bachelor degree year 1/2/3/4/<br>Diploma year 1/2/3/4
**12**	Other tertiary educational background ? (provide details on institution, program, and whether completed or not)	
**13**	What future job / role are you looking to fulfill in your career / work life?	
**14**	In your own words what does animal welfare mean to you?	
**15**	What is the importance of animal welfare in Vietnamese culture and society according to your understanding?	Animal welfare is not important for any animals in Vietnam/ Animal welfare is not important for aquatic animals in Vietnam, but important for non-aquatic animals/<br>AAW has some importance in Vietnam, but I am unsure of the importance/<br>AAW is extremely important in Vietnam /<br>AAW has mostly not been important previously, but is now increasing in importance in Vietnam/<br>I do not know.
**16**	In your opinion which aspects contribute to good AAW? Tick all that apply	Adequate space for natural behaviour/

		Respect of species specific behaviours and instincts; Consideration of social needs and interactions with its own kind/ Access to good water quality and a suitable habitat/ Protection from pain, injury, and disease/ Access to veterinary care and medical attention when needed/ Proper nutrition and feeding practices/ Opportunities for mental stimulation/ Reduced stress factors/ I do not have enough knowledge to answer/ Other
**17**	Where do you think animal welfare is an important feature when farming aquatic animals? Tick all that apply	Handling / transportation/ harvest/ slaughter/ general husbandry/ I do not have enough knowledge to answer / other
**18**	Do you eat aquatic animals (fish or shellfish) and how often?	No/ daily/ at least once a week/ several times a week/ at least once a month/ yes, not often.
**19**	(i) If you had the choice of buying fresh fish or shellfish from your local supermarket which of the products below would you choose?	Product A (fresh, locally farmed (intensive) (300g), clear air-tight sealed packaging, ~65 000 VND)/ Product B (the same as product A but which is sourced from a farm practising high welfare standard in their husbandry, harvesting, and slaughter of animals)/ it makes no difference to me, so either product.
**19**	(ii) Would you pay extra for product B? *	No/ yes
**19**	(iii) If you answered yes to the previous question, how much more would you pay for product B (in %) and why?	
**20**	In your opinion AAW is an important consideration for: (select all that apply)	Farming/ owning an aquatic pet/ conducting research with aquatic animals/ fulfilling regulations and policies in Vietnam/ fulfilling

		requirements involved with international trade in aquatic animal commodities/ I do not know
**21**	Rate your confidence level in discussing the following components of aquatic animal welfare. Select one answer from each row.	Categories: nutrition and feeding/ pain/ health and disease/ natural behaviour/ habitat needs/ emotional states (fear, distress, etc.); environmental enrichment/ ethics / social values Options: score 1-5 (not confident – very confident)
**22**	On a scale of 1-5 how important is learning about AAW to your academic and professional interests?	Score 1-5 (very unimportant – very important)
**23**	Do you feel you are taught enough about AAW in your current curriculum?	Yes/ no/ not sure/ it is of no interest to me/ not applicable since I have not learned about this.
**24**	How would you describe your satisfaction with study materials, assignments, resources available, and overall learning experience on AAW in your current program?	Very dissatisfied/ dissatisfied/ neither dissatisfied nor satisfied/ satisfied/ very satisfied/ not sure/ not applicable since I have not learned about this (*Vietnamese version did not reflect the English version)
**25**	How would you describe your satisfaction with your instructors teaching on AAW with respect to quality of teaching, communication skills, availability for support, and level of knowledge?	Very dissatisfied/ dissatisfied/ neither dissatisfied nor satisfied/ satisfied/ very satisfied/ not sure/ not applicable since I have not learned about this (*Vietnamese version did not reflect the English version)
**26**	What changes, if any, are you interested in seeing in your current program concerning AAW (theoretical and practical parts) ?	
**27**	Do you want to include AAW in your training program? If yes, go to question 28. If no, go to question 29.	
**28**	(i) How do you want the topic to be included? Tick all that apply.	A new subject on AAW/ integrate it into current technical subjects/ practical experience programs at a company/ talks, seminars, workshops with specialists/ others
**28**	(ii) If choosing "Other", please specify.	
**29**	Would education in AAW influence your future job prospects	

in any way (locally or internationally)? Why?

Following question was accidentally excluded in the translated version: Have you heard about AAW issues and why they matter,
outside of your university / college studies? Select all that apply

Table S4: Survey questions for the perception study on industry stakeholders within South and South-Central Vietnam. Questions in bold were compulsory. Where options are provided for certain questions, these are highlighted in the options column. Questions shaded in grey are welfare scoring questions.

No.	Questions	Options
**1-3**	Full name; email address; phone number	
**4**	Please indicate your gender?	Male/ female/ other/ prefer not to say
**5**	Please indicate your age range?	25-35 / 36-45/ 46-55/ 56-65/ >65
**6**	What religion do you identify with?	Buddhism/ Taoism/ Christian/ Atheist or agnostic/ other
**7**	What nationality are you?	
**8**	How much is the monthly household income of the head of your household in VND (Vietnamese Dong)? (this may be yourself or another person)	<30 000 000/ 20 000 000-30 000 000/ 10 000 000-20 000 000/ <10 000 000
**9**	What is the name of the institution you represent?	
**10**	What is your current role / position at the institution?	
**11**	What other roles /positions have you worked before this?	
**12**	What is your educational background?	
**13**	In your own words what does animal welfare mean to you?	
**14**	What is the importance of animal welfare in Vietnamese culture and society according to your understanding?	Animal welfare is not important for any animals in Vietnam/Animal welfare is not important for aquatic animals in Vietnam, but important for non-aquatic animals/ AAW has some importance in Vietnam, but I am unsure of the importance/ AAW is extremely important in Vietnam / AAW has mostly not been important previously, but is now increasing in importance in Vietnam/ I do not know.
**15**	In your opinion which aspects contribute to good AAW? Tick all that apply.	Adequate space for natural behaviour/ Respect of species specific behaviours and instincts; Consideration of social needs and interactions with its own kind/

		Access to good water quality and a suitable habitat/<br>Protection from pain, injury, and disease/<br>Access to veterinary care and medical attention when needed/<br>Proper nutrition and feeding practices/<br>Opportunities for mental stimulation/<br>Reduced stress factors/<br>I do not have enough knowledge to answer/<br>Other
**16**	Where do you think animal welfare is an important feature when farming aquatic animals? Tick all that apply	Handling / transportation/ harvest/ slaughter/ general husbandry/ I do not have enough knowledge to answer / other
**17**	Do you eat aquatic animals (fish or shellfish) and how often?	No/ daily/ at least once a week/ several times a week/ at least once a month/ yes, not often.
**18**	(i) If you had the choice of buying fresh fish or shellfish from your local supermarket which of the products below would you choose?	Product A (fresh, locally farmed (intensive) (300g), clear air-tight sealed packaging, ~65 000 VND)/<br>Product B (the same as product A but which is sourced from a farm practising high welfare standards in their husbandry, harvesting, and slaughter of animals)/<br>it makes no difference to me, so either product.
**18**	(ii) Would you pay extra for product B? *	No/ yes
**18**	(iii) If you answered yes to the previous question, how much more would you pay for product B (in %) and why?	
**19**	On a scale of 1-5 how important do you think it is for students who are involved in aquaculture, veterinary, and aquatic animal health-related professions to understand AAW and how this applies to relevant industries?	Score 1-5 (very unimportant-very important)
**20**	(i) Can the staff recruited, who are university or college graduates, identify issues related to AAW?	Yes/ no<br>*the options used in the Vietnamese version differed from the English version (i.e. not fully trained, maybe, cannot yet)

**20**	(ii) If yes, how do you know that they can?	
**21**	On a scale of 1 - 5 how willing are you to support tertiary institutions to help optimise their learners' experience in AAW?	Score 1-5 (very unwilling- very willing)
**22**	Is your company able to make a formal agreement with a tertiary institution to offer support to learners in AAW training?	Score 1-5 (very unwilling- very
**23**	What sort of support would you be interested in offering ? Tick all that apply	Technical/ financial/ use of facilities/ use of animals/ other
**24**	Is there any other level of support to tertiary institutions that you can offer to learners, or graduates who may be interested in continual professional development (CPD) on AAW?	
**25**	Are there specific areas within industry where you would like to see graduates add more value? Tick all that apply	Welfare research/ Policy and legislation development (public or private sector)/ Industry practices (any facility that uses aquatic animals for any purpose)/ Public awareness and advocacy/ Conservation/ Aquaculture/ Other

Table S5: Summary of scoring questions from the perception survey indicating options available to respondents and scoring for each.

Questions	Options	Scoring
In your own words what does animal welfare mean to you?		
What is the importance of animal welfare in Vietnamese culture and society according to your understanding?	Animal welfare is not important for any animals in Vietnam	0
	Animal welfare is not important for aquatic animals in Vietnam, but important for non-aquatic animals	4
	AAW has some importance in Vietnam, but I am unsure of the importance	6
	AAW is extremely important in Vietnam	10
	AAW has mostly not been important previously, but is now increasing in importance in Vietnam	8
	I do not know	2
In your opinion which aspects contribute to good AAW? Tick all that apply	Adequate space for natural behaviour/	1 point /option selected
	Respect of species-specific behaviours and instincts;	
	Consideration of social needs and interactions with its own kind/	
	Access to good water quality and a suitable habitat/	
	Protection from pain, injury, and disease/	
	Access to veterinary care and medical attention when needed/	
	Proper nutrition and feeding practices/	
	Opportunities for mental stimulation/	
	Reduced stress factors/	
	I do not have enough knowledge to answer/	
	Other	

Where do you think animal welfare is an important feature when farming aquatic animals? Tick all that apply.	Handling / transportation/ harvest/ slaughter/ general husbandry/ I do not have enough knowledge to answer / other	1 point /option selected
(i) If you had the choice of buying fresh fish or shellfish from your local supermarket which of the products below, would you choose?	Product A: fresh, locally farmed (intensive) (300g), clear air-tight sealed packaging (~65 000 VND)	5
	Product B: the same as product A but which is sourced from a farm practicing high welfare standard in their husbandry, harvesting, and slaughter of animals	10
	It makes no difference to me, so either product	0
(ii) Would you pay extra for product B?	No	0
	Yes	5
Questions	Options	Scoring
On a scale of 1-5 how important do you think is the inclusion of AAW in the education of learners in aquaculture and animal health professions?	1 -very unimportant	2
	2- unimportant	4
	3- neutral	6
	4- important	8
	5- very important	10
On a scale of 1-5 how important is learning about AAW to your academic and professional interests?	1 -very unimportant	2
	2- unimportant	4
	3- neutral	6
	4- important	8
	5- very important	10
On a scale of 1-5 how important do you think it is for students who are involved in aquaculture, veterinary, and aquatic animal health-related	1 -very unimportant	2
	2- unimportant	4

professions to understand AAW and how this applies to relevant industries?	3- neutral	6
	4- important	8
	5- very important	10
On a scale of 1 - 5 how willing are you to support tertiary institutions to help optimise their learners' experience in AAW?	1 -very unwilling	2
	2- unwilling	4
	3- neutral	6
	4- willing	8
	5- very willing	10
In your opinion AAW is an important consideration for: (select all that apply)	Farming/ owning an aquatic pet/ conducting research with aquatic animals/ fulfilling regulations and policies in Vietnam/ fulfilling requirements involved with international trade in aquatic animal commodities/ I do not know	1 point/ option

Table S6 : Summary of questions for the education gap score indicating options available to respondents and scoring used.

Questions	Options	Gap score
Rate your confidence level in discussing the following components of aquatic animal welfare. Select one answer from each row. [scores were added together for each component and designated into 4 categories-see options column]	No confidence	10
	Slightly confident	7.5
	Somewhat confident	5
	Fairly to very confident	2.5
Do you feel you are taught enough about AAW in your current curriculum?	Yes	2
	No	10
	Not sure	6
	It is of no interest to me	8
	Not applicable since I have not learned about this.	4
How would you describe your satisfaction with study materials, assignments, resources available, and overall learning experience on AAW in your current program?	Very dissatisfied	10
	No interest	8.35
	Relatively satisfied	3.34
	Very satisfied	1.67
	Not sure	6.68
	Not applicable since I have not learned about this	5.01

How would you describe your satisfaction with your instructors teaching on AAW with respect to quality of teaching, communication skills, availability for support, and level of knowledge?	Very dissatisfied	10
	Relatively satisfied	4
	Very satisfied	2
	No interest	8
	Not applicable	6
